# A Prospective Randomized Comparison of Postoperative Pain and Complications after Thyroidectomy under Different Anesthetic Techniques: Volatile Anesthesia versus Total Intravenous Anesthesia

**DOI:** 10.1155/2021/8876906

**Published:** 2021-02-02

**Authors:** Jun-Young Jo, Yeon Ju Kim, Seong-Soo Choi, Jihoon Park, Han Park, Kyung-Don Hahm

**Affiliations:** Department of Anesthesiology and Pain Medicine, Laboratory for Perioperative Outcome Research, Asan Medical Centre, University of Ulsan College of Medicine, 88 Olympic-ro 43-gil, Songpa-gu, Seoul 05505, Republic of Korea

## Abstract

While the postoperative outcome is favorable, post-thyroidectomy pain is considerable. Reducing the postoperative acute pain, therefore, is considered important. This study investigated whether the pain intensity and need for rescue analgesics during the immediate postoperative period after thyroidectomy differ according to the methods of anesthesia. Seventy-two patients undergoing total thyroidectomy under general anesthesia were examined. Patients were randomly assigned to undergo either total intravenous anesthesia with remifentanil and propofol (TIVA, *n* = 35) or propofol induction and maintenance with desflurane and nitrous oxide (volatile anesthesia [VA], *n* = 37). The mean administered dose of remifentanil was 1977.7 ± 722.5 *μ*g in the TIVA group, which was approximately 0.268 ± 0.118 *μ*g/min/kg during surgery. Pain scores based on a numeric rating scale (NRS) and the need for rescue analgesics were compared between groups at the postoperative anesthetic care unit (PACU). The immediate postoperative NRS values of the TIVA and VA groups were 5.7 ± 1.7 and 4.7 ± 2.3, respectively (*P* = 0.034). Postoperative morphine equianalgesic doses in the PACU were higher in the TIVA group than in the VA group (16.7 ± 3.8 mg vs. 14.1 ± 5.9 mg, *P* = 0.027). The incidence of immediate postanesthetic complications did not differ significantly between groups. In conclusion, more rescue analgesics were required in the TIVA group than in the VA group to adequately manage postoperative pain while staying in the PACU after thyroidectomy.

## 1. Introduction

Thyroid surgeries are considered to be safe operations with a relatively low level of morbidity and mortality [1–3]. Nevertheless, patients suffer from quite severe immediate postoperative pain [4]. Since inappropriate acute pain control could lead to chronic pain and even increased opioid consumption, which is treated as a social problem in extreme cases [5], clinicians should try to reduce acute postoperative pain.

The previous propensity-score matching analysis, which we retrospectively conducted on 7,511 thyroidectomy patients [6], showed comparable results. In particular, general anesthesia with continuous remifentanil infusion was related with a higher intensity of immediate postoperative pain than that without remifentanil infusion. The method of general anesthesia could have an effect on immediate postoperative pain as well as medications used to directly manage postoperative pain. Although this was a fairly large-scale study, there were limitations due to several uncontrolled conditions derived from the innate nature of a retrospective study. Among them, the most concerning uncontrolled aspects were the type of hypnotics used during maintenance of general anesthesia and how postoperative pain was controlled.

Total intravenous anesthesia (TIVA) refers to an anesthetic method in which general anesthesia is maintained by intravenous drug infusion rather than by inhalant agents. The hypnotic agent commonly used in TIVA is propofol, and opioids are added for an analgesic effect. TIVA is particularly useful in some situations where inhalational anesthesia is contraindicated or may interfere with the operation. Several recent studies also showed that TIVA leads to better outcomes and even relieves postoperative pain compared with inhalant anesthesia [7, 8]. Nevertheless, many anesthesiologists prefer volatile anesthesia (VA) because it is relatively easy to use, especially in short-duration surgeries.

Therefore, in this current randomized controlled study, we aimed to investigate the degree of postoperative pain in two methods of anesthesia: TIVA with continuous infusion of propofol and remifentanil and VA with only inhalant agents. Furthermore, other postanesthetic complications were also evaluated as secondary outcomes.

## 2. Materials and Methods

### 2.1. Ethical Approval and Study Population

We obtained approval from the Institutional Review Board of Asan Medical Centre, Seoul, Korea (2016–0463), and registered this study at cris.nih.go.kr (KCT0005047). All participants gave a written informed consent. We included 80 patients. The inclusion criteria were as follows: an age range of 20 to 79 years, either sex, having an American Society of Anesthesiologists Physical Status I–III, and undergoing an elective thyroid surgery by the same surgeon. The exclusion criteria were as follows: allergy or contraindication to opioids or nonsteroidal anti-inflammatory agents (NSAIDs); severe renal dysfunction with limited use of NSAIDs; bronchial asthma; psychiatric disease; intake of opioids within one month before surgery or any analgesics 6 h before surgery; reoperation on thyroid; or simultaneous radical neck lymph node dissection or other surgeries.

### 2.2. Study Design and Blindness

This study was a prospective, assessor-blinded, randomized controlled study. Because of the significant differences between the two anesthetic techniques, the attending anesthesiologist could not be blinded to group identity. However, the patient and outcome assessors were blinded to group identity.

Subjects were randomly assigned in a 1 : 1 ratio to two groups using an online randomization program (http://www.randomization.com). Randomization was done the day before surgery, and each patient was randomly assigned to the TIVA or VA group. Standard monitoring included noninvasive blood pressure, heart rate, electrocardiogram, and a bispectral index (BIS) monitor (BIS-Vista™ monitors; Aspect Medical Systems, Newton, MA).

Regardless of the assigned group, all patients were induced with general anesthesia with 1.5∼2.5 mg/kg propofol and 0.6 mg/kg rocuronium. Patients assigned to the TIVA group were maintained on anesthesia with an effect-site target-controlled infusion (TCI) of propofol and remifentanil using a commercial TCI pump (Orchestra® Base Primea; Fresenius Vial, Brezins, France). The effect-site concentration of propofol was controlled between 2.0 and 2.5 *μ*g/mL to maintain BIS of 40 to 60 and that of remifentanil was titrated to maintain the patient's baseline blood pressure within 20%. In the VA group, general anesthesia was maintained with desflurane and 50% nitrous oxide (N_2_O) in oxygen, and the concentration of desflurane was controlled to maintain the patient's baseline blood pressure within 20%, which was approximately 1 minimum alveolar concentration of desflurane. At the end of surgery, the anesthetic agents were immediately discontinued. After enough oral suctioning, the inhaled oxygen fraction and fresh gas flow rate were increased to 100% and 8 L, respectively, in order to facilitate emergence. Neuromuscular blocking was reversed with 0.4 mg glycopyrrolate and 15 mg pyridostigmine and confirmed by a train-of-four monitor. Endotracheal tubes were removed when patients regained consciousness with spontaneous respiration. Patients were then transferred to the postoperative anesthetic care unit (PACU) and scored for pain.

Pain management at the PACU was performed in the same manner in both groups. If the pain score was over 5, the anesthesiologists ordered intravenous rescue analgesics. The first rescue analgesic was an NSAID, which was 30 mg ketorolac. After 15 min, the pain score was reevaluated, and the second rescue analgesic medication administered was 50 *μ*g fentanyl if the pain score was still over 5. At 30 min after entering the PACU, the pain score was evaluated again, and when exceeding 5, the previously administered opioid was readministered in the same amount.

### 2.3. Outcome Assessment

The primary outcome was the pain intensity assessed at the PACU at predefined time points: upon arrival to PACU, 30 min after surgery, and 60 min after surgery. This was assessed by experienced nurses who were blinded and independent of the study based on numeric rating scales (NRS) at the PACU. The NRS scale was an 11-point numerical rating scale (0 = no pain; 10 = unbearable pain). Simultaneously, the amount of rescue analgesics administered to manage postoperative pain was recorded. Morphine equianalgesic doses (MED) were used to quantitatively compare the effects of the sum of various rescue analgesics (fentanyl or ketorolac) administered to each group. The conversion to morphine-equivalent consumption in mg was based on the following scale: 1 : 100 for fentanyl; 1 : 0.4 for ketorolac [9, 10].

From the induction of general anesthesia to skin incision, the patient's blood pressure, heart rate, and BIS were recorded at five predefined time points: before induction of general anesthesia (T1), before intubation (T2), 1 min after intubation (T3), before skin incision (T4), and 1 min after skin incision (T5). Incidences of postanesthetic complications such as postoperative nausea and vomiting (PONV), itching or urticaria, shivering, and desaturation due to respiratory depression were also evaluated as secondary outcomes.

### 2.4. Sample Size Calculation and Statistical Analysis

To calculate sample size, we used G *∗* Power 3.1.9.4. According to a previous retrospective study, the difference of the NRS score immediately after surgery was 1.58 between two groups with or without remifentanil during thyroidectomy, and the standard deviations of these groups were 2.610 and 1.706, respectively [6]. Thus, we originally needed 64 subjects (32 in each group) to achieve a study power of 80% and detect a difference in score of 1.58 using a two-sided Student's *t*-test at a significance level of 0.05. Finally, a total of 80 subjects (40 in each group) were needed assuming a 20% dropout rate.

All continuous variables are expressed as means ± standard deviations and categorical variables are expressed as frequencies and percentages. To compare perioperative data, an unpaired, two-tailed *t*-test was used for continuous variables, and a chi-squared test was used for categorical variables. Data manipulation and statistical analysis were performed using SPSS Statistics for Windows, Version 21.0 (IBM; Armonk, NY). All reported *P* values are two-sided, and *P* values < 0.05 were considered statistically significant.

## 3. Results and Discussion

### 3.1. Results

**Figure 1:**
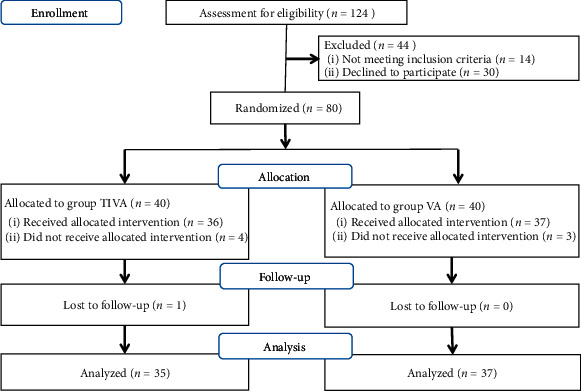
CONSORT diagram of participant flow.

During the entire trial, a total of 124 patients were planned to undergo thyroidectomy, but 14 were excluded because they did not meet the inclusion criteria, and 30 refused to participate in the trial. Finally, 80 patients were enrolled (Figure 1). One patient in the TIVA group was reopened due to bleeding in the wake of anesthesia, and seven patients (four from the TIVA group; three from the VA group) were excluded from the analysis because rescue analgesic administration in the recovery room was not the same as planned. Finally, data from 72 patients was used for analysis.

**Figure 2:**
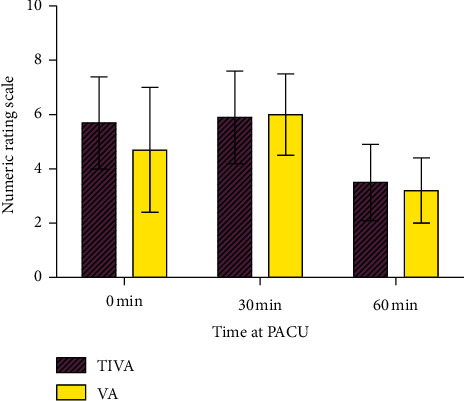
Pain intensity at the postoperative anesthetic care unit according to time from arrival. PACU = postoperative anesthetic care unit.

Table 1 shows that the demographic data and basal characteristics were not different between groups. The TIVA and VA anesthetic durations were 91.0 ± 24.7 min and 89.5 ± 20.2 min, respectively (*P* = 0.776). The mean administered dose of remifentanil was 1977.7 ± 722.5 *μ*g in the TIVA group, which was approximately 0.268 ± 0.118 *μ*g/min/kg during surgery. The comparison of pain intensity over time evaluated at the PACU is shown in Figure 2. The first NRS score assessed upon arrival at the PACU before administration of rescue analgesics was higher in the TIVA group than that in the VA group (5.7 ± 1.7 vs. 4.7 ± 2.3; *P* = 0.034). The second and third assessed pain intensities in both groups did not show statistically significant differences. The second and third NRS scores in the TIVA group were 5.9 ± 1.7 and 3.5 ± 1.4, and those in the VA group were 6.0 ± 1.5 and 3.2 ± 1.2 (*P* = 0.824 and *P* = 0.294, resp.). The mean NRS scores of maximal pain intensity, while staying at the PACU, of the TIVA and VA groups were 6.8 ± 1.0 and 6.5 ± 1.4, respectively (*P* = 0.187). As shown in Table 2, all patients in the TIVA group received rescue analgesics at the PACU. However, 89.2% of patients in the VA group needed rescue analgesics. The doses for rescue analgesics at the PACU are shown in Table 2. Intravenous NSAIDs were administered more in the TIVA group (30.0 ± 0.0 mg vs. 26.8 ± 9.4 mg; *P* = 0.044), but the administered dose of fentanyl between groups was not significantly different (*P* = 0.111). The sum of rescue analgesics administered to the TIVA group after conversion to MED was significantly higher than that of the VA group (16.7 ± 3.8 mg vs. 14.1 ± 5.9 mg; *P* = 0.027).

**Figure 3:**
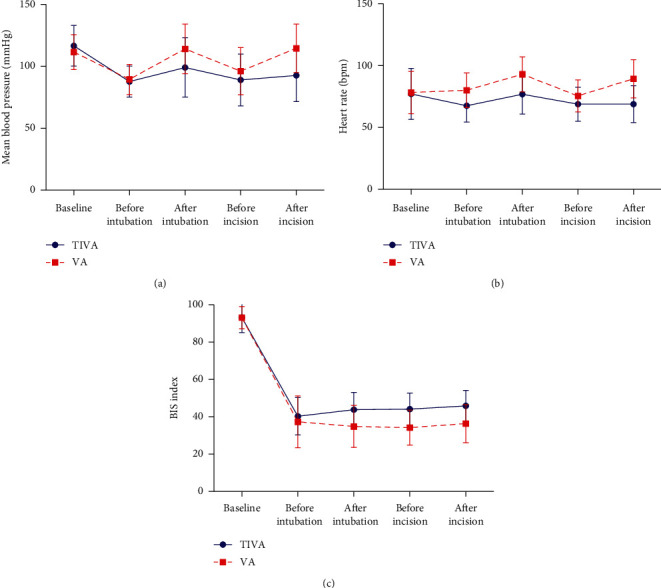
The changes in mean blood pressure, heart rate, and bispectral (BIS) index during surgery. TIVA = total intravenous anesthesia; VA = volatile anesthesia.

During induction and before and after skin incision, the BIS of both groups was maintained between 30 and 60, but that of the TIVA group was sustained at a higher level than that of the VA group (Figure 3(c)). The TIVA group showed fewer changes in mean blood pressure and heart rate than the VA group (Figures 3(a) and 3(b)). PONV was the most common postoperative complication in both groups. The incidences of PONV in the TIVA and VA groups were 42.9% and 43.2, respectively, which were not significantly different. Additionally, other complications including itching or urticaria and shivering were not significantly different between groups (Table 3).

### 3.2. Discussion

The main finding in this prospective, assessor-blind, randomized controlled study was that the intensity of immediate postoperative pain was higher in the TIVA group than the VA group before any analgesics were administered. Furthermore, the demand for rescue analgesics, during PACU stay, in the TIVA group was higher than that in the VA group for effectively managing postoperative pain. In addition, there was no difference in the incidence of complications immediately after surgery.

Rapid recovery and emergence from general anesthesia are important in cases of ambulatory surgery or enhanced recovery after surgery. Both TIVA and VA are popular and reasonable for relatively simple surgeries, which are usually performed as ambulatory surgeries. Volatile agents have become more popular in general anesthesia since sevoflurane and desflurane were introduced because they allow for faster recovery [11]. Propofol, one of the intravenous anesthetics, is also frequently used in ambulatory surgery with the continuous infusion of remifentanil, which has become more popular after the introduction of the TCI pump [12]. TIVA and VA have been developing side-by-side, and either anesthetic method, if not contraindicated, is often chosen according to the anesthesiologist's preference and the institutional circumstances. In this current study, we compared the pain intensities after general anesthesia with two representative anesthetic methods that are suitable for relatively simple and minor surgeries.

The pain assessment was performed three times every 30 min. The first one is differentiated from the others because enough rescue analgesics were administered during PACU stay. The first NRS score represents analgesic effects depending on the anesthetic method, and the others show the degree of controlled immediate postoperative pain. According to the protocol, NSAIDs were first administered as a rescue analgesic agent at the PACU, and opioids were administered additionally. In order to analyze the demands of the two groups in maintaining adequate pain control, the sum of MED rescue analgesics, including both NSAIDs and opioids, was compared. Although the difference was not obvious, patients in the TIVA group complained of a more immediate postoperative pain compared with patients in the VA group. Moreover, the TIVA group needed more rescue analgesics than the VA group during the recovery period in order to maintain sufficient pain control. These results contradict those of a meta-analysis of randomized controlled studies [13, 14]. Moreover, in a randomized controlled study conducted by Wong et al. [15], patients who underwent colorectal surgery did not show any difference in pain intensity between TIVA and VA. However, some differences might be present, such as the precise anesthetic methods, total anesthetic duration, site and size of surgical wound, and degree of tissue injuries. The VA group was administered two types of volatile agents: N_2_O and desflurane. Meanwhile, remifentanil was the only drug that gave an analgesic effect in the TIVA group. Although we did not check the sedative level during the recovery phase, residual VA could result in lower NRS scores in the VA group. Other possible causes for higher NRS scores in the TIVA group might include opioid-induced hyperalgesia (OIH) or acute opioid tolerance (AOT). The exact mechanisms of these two phenomena are unclear, and some cellular- and molecular-level explanations have been suggested [16]. According to a systematic review by Kim et al. [17], the incidence of OIH and AOT is dose-dependent, and administering more than 2.7 ng/mL using a TCI pump and 0.1 *μ*g/kg/min with continuous infusion of remifentanil could lead to OIH and AOT [18–24]. We used a TCI pump in this study, and the total intraoperative administered dose of remifentanil estimated was regarded as enough to lead to OIH and AOT, based on previous studies.

Remifentanil has the advantage of controlling vital signs with an ultrashort action time. Despite its relatively high cost and the need to set up a special machine for TCI, many clinicians prefer anesthesia by remifentanil infusion in order to maintain hemodynamic stability during surgery [25, 26]. We titrated the concentration of the volatile agent to systolic blood pressure within 20% of the baseline, but the control ability of remifentanil in the TIVA group was superior to volatile agents in the VA group. During and after inducing general anesthesia, the BIS in the VA group remained lower than the TIVA group, inferring that deeper anesthesia was achieved. Nevertheless, vital signs of subjects in the VA group responded significantly to noxious stimulations such as intubation and skin incision, which suggests that the autonomic nervous system was not adequately suppressed by volatile agents alone.

In this randomized controlled study, we did not observe differences in the incidence of postoperative complications including PONV, shivering, itching, or respiratory complications between the groups. This is inconsistent with a meta-analysis by Schraag et al. [13] that showed that the risk for PONV was lower with propofol and remifentanil than with inhalational agents. This might be because our study was performed in cases of minor surgery with relatively less pain and shorter operation times, so the amounts of anesthetic and opioids used were small. Therefore, for complications following general anesthesia, including PONV, accurate results need to be obtained through studies conducted with more controlled variables and subjects.

There are several limitations in this study. First, we cannot explain the hyperalgesia after surgery which was induced only by intraoperative continuous infusion of remifentanil. In this study, propofol in the TIVA group and desflurane in the VA group were used as sedatives. The perception of postoperative pain intensity could be affected by the different sedatives in each group. To confirm that remifentanil induced OIH, the study should have been conducted with the same sedative in both groups. However, in this study, we aimed to compare the two anesthetic methods using the same sedatives which do not have any analgesic effect. In addition, we did not focus on chronic pain transition. The intensity and duration of acute postoperative pain is known to be a predictor of chronic postsurgical pain [27]. If patients are followed in the long-term, we could find the incidence of transition from acute to chronic pain.

## 4. Conclusions

General anesthesia with TIVA could lead to a higher but clinically acceptable intensity of immediate postoperative pain with more rescue analgesics for proper pain control. Moreover, there was no difference in the incidence of postoperative complications between the two methods. Therefore, in the case of minor surgery, with relatively little pain involved and no major bleeding expected, anesthesiologists may choose between TIVA and VA depending on the level of technical expertise and availability of resources at each institution.

## Figures and Tables

**Table 1:** Demographics of the study population and basal characteristics.

	TIVA group (*n* = 35)	VA group (*n* = 37)	*P* value
Age (year)	49.3 ± 13.5	48.4 ± 11.5	0.752
Male	7 (20.0)	9 (24.3)	0.659
Weight (kg)	65.3 ± 12.7	63.1 ± 11.3	0.431
Height (cm)	159.8 ± 7.0	161.0 ± 8.0	0.505
Body mass index (kg/m^2^)	25.5 ± 4.1	24.2 ± 2.8	0.123
Comorbidities			
Diabetes mellitus	3 (8.6)	2 (5.4)	0.670
Hypertension	13 (37.1)	10 (27.0)	0.358
Smoking^*∗*^	5/4 (14.3/11.4)	1/2 (2.7/5.4)	0.114
Alcohol	11 (31.4)	12 (32.4)	0.927
Vital signs at ward in the morning of surgery			
Systolic blood pressure	120.6 ± 14.9	117.2 ± 14.8	0.336
Diastolic blood pressure	76.7 ± 8.5	72.3 ± 14.8	0.132
Heart rate	75.2 ± 11.6	75.8 ± 12.5	0.831
Anesthetic duration (min)	91.0 ± 24.7	89.5 ± 20.2	0.776

TIVA = total intravenous anesthesia; VA = volatile anesthesia. ^*∗*^Smoking: exsmoker/current smoker. Data are represented with mean ± SD for continuous variables and with number (%) for categorical variables.

**Table 2:** Demands for rescue analgesics administered intravenously during stay at the postoperative anesthetic care unit.

	TIVA group (*n* = 35)	VA group (*n* = 37)	*P* value
Need for rescue analgesics			
NSAID	35 (100)	33 (89.2)	0.115
Fentanyl	24 (68.6)	21 (56.7)	0.155
Once	14 (40.0)	17 (45.9)	
Twice	10 (28.6)	4 (10.8)	
Doses for rescue analgesics			
NSAID (mg)	30.0 ± 0.0	26.8 ± 9.4	0.044
Fentanyl (*μ*g)	47.4 ± 38.3	33.8 ± 33.4	0.111
MED (mg)	16.7 ± 3.8	14.1 ± 5.9	0.027

NSAID = nonsteroidal anti-inflammatory drug; MED = morphine equianalgesic dose; TIVA = total intravenous anesthesia; VA = volatile anesthesia. Data are represented with mean ± SD for continuous variables and with number (%) for categorical variables.

**Table 3:** Occurrence of postoperative complications.

	TIVA group (*n* = 32)	VA group (*n* = 35)	*P* value
PONV	15 (42.9)	16 (43.2)	0.974
Itching or urticaria	0 (0.0)	1 (2.7)	1.000
Shivering	2 (5.7)	1 (2.7)	0.609
Desaturation	0 (0.0)	0 (0.0)	—

PONV = postoperative nausea and vomiting; TIVA = total intravenous anesthesia; VA = volatile anesthesia. Data are represented with number (%) for categorical variables.

## Data Availability

The data used to support the findings of this study are restricted by the Institutional Review Board of Asan Medical Centre, Seoul, Korea, in order to protect patient privacy. Data are available from the corresponding author for researchers who meet the criteria for access to confidential data.
